# Psychometric assessment of the EMIC Stigma Scale for Brazilians affected by leprosy

**DOI:** 10.1371/journal.pone.0239186

**Published:** 2020-09-17

**Authors:** Fabiane Frota da Rocha Morgado, Erika Maria Kopp Xavier da Silveira, Lilian Pinheiro Rodrigues do Nascimento, Anna Maria Sales, José Augusto da Costa Nery, Euzenir Nunes Sarno, Ximena Illarramendi

**Affiliations:** 1 Department of Physical Education and Sports, Federal Rural University of Rio de Janeiro, Rio de Janeiro, Brazil; 2 Souza Araújo Outpatient Clinic, Leprosy Laboratory, Oswaldo Cruz Institute, Oswaldo Cruz Foundation, Rio de Janeiro, Brazil; 3 Center for Technological Development in Health, Oswaldo Cruz Foundation, Rio de Janeiro, Brazil; Monash University, AUSTRALIA

## Abstract

**Background:**

The Stigma Scale of the Explanatory Model Interview Catalogue (EMIC-SS) is a useful option to investigate leprosy-related stigma, but its psychometric qualities are unknown in Brazil. This study investigated the factor structure, the convergent and known-groups validity, and the reliability of the EMIC-SS for Brazilians affected by leprosy.

**Methodology:**

The Brazilian Portuguese version of the EMIC-SS was validated in 180 persons affected by leprosy at a Reference Center in Rio de Janeiro. Confirmatory factorial analysis (CFA) and Cronbach alpha were used to assess the EMIC-SS internal consistency. The Construct validity was tested using Spearman Correlation, Kruskal-Wallis, and Mann-Whitney tests comparing with the Participation Scale, Rosenberg Self-esteem Scale, Beck Depression Inventory, and a Sociodemographic Questionnaire. Test-retest reliability was evaluated with intra-class correlation (ICC).

**Main findings:**

CFA confirmed the one- and two-dimensional models of the scale after retaining 12 of the 15 EMIC-SS items. The 12—item EMIC-SS was consistent (α = 0.78) and reproducible (ICC = 0.751, 95% Confidence Interval = 0.657–0.822, p < 0.0001). A significant correlation was observed between the EMIC-SS and the other scales confirming convergent validity. The EMIC-SS and its factors were able to differentiate several hypothesized groups (age, change of occupation, monthly family income, communicating others about the disease, and perception of difficulty to follow treatment) confirming the scale known-groups validity, both in its one and two-dimensional models.

**Conclusions/Significance:**

Our study found support for the construct validity and reliability of the EMIC-SS as a measure of stigma experienced by people affected by leprosy in Brazil. However, future studies are necessary in other samples and populations with stigmatizing conditions to determine the optimal factor structure and to strengthen the indications of the validated scale.

## Introduction

The past decade has been important for developing the new science of stigma, a fundamental social cause of health inequalities. The paradigm of stigma has changed and today it is viewed as a cultural disease. It affects processes such as resource availability, social relationships, psychological, and behavioural responses, all of which mediate adverse population health outcomes [1]. At the individual level, the exclusion, rejection, and guilt or devaluation sensation resulting from the experience, perception, or anticipation of an adverse social judgment [2], might affect the health outcome of the stigmatized person. This judgment is based on a long-life characteristic of the identity conferred to a specific health problem, a condition, or social aspects [3].

Usually, stigma frameworks focus on specific diseases in isolation [4]. But leprosy, unlike other stigmatized diseases, is a composite of different conditions. It is a chronic infectious disease that mainly affects the skin and the peripheral nerves, which may be aggravated during the immunoinflammatory episodes known as leprosy reactions. The presence of overt skin lesions, physical disability, and deformities, all of which may remain even after release from treatment [5], can all lead to discrimination. Moreover, leprosy affects the most deprived individuals in society [6]. Leprosy has diverse social, economic, cultural, and political dimensions amassed into a deeply stigmatized social category that, throughout history, as well as in contemporary societies, has been used mainly to exclude the affected people [7]. Thus, the stigma experience of persons affected by leprosy might be amplified due to intersecting stigmas, which need to be addressed when studying and understanding the stigma framework in leprosy.

Leprosy-related stigma has been the most persistent and immanent form of prejudice, social injustice, and discrimination that societies have inflicted on human beings throughout history. It adversely impacts the psychological and emotional wellbeing of the affected people, who are denied their most basic human rights. Being unjustly blamed for their disease, or feeling excluded or punished for supposed wrongdoing, the people affected by leprosy suffer from low self-esteem, fear, shame, guilt, anxiety, hopelessness, anger. As expected, this has damaging effects in various life domains, leading to self-isolation and thus, withdrawal from work and social life. At the societal level, it undermines social relationships, enabling rejection, forced isolation, and restricted social participation [8]. Finally, leprosy-related stigma is a major contributor to the burden of illness and may influence effective case finding and treatment, which are key elements for disease control. In Brazil, the long history of involuntary isolation of individuals affected by leprosy, added to years of beliefs, myths, and misconceptions regarding its cause, transmission, treatment, and consequences [9–11], have left a profound scar in our society. The Brazilian government has strived to reduce leprosy-related stigma and to improve social integration. An initial attempt was by changing the term leprosy to hanseniasis, initiated during the 1970s, and officially recognized by federal law in 1995 [12].

Leprosy control measures to reduce transmission and stigma have been mainly directed towards early diagnosis and adequate treatment to prevent disabilities, as well as health education, and assessment of activity limitation and social participation [13]. However, to only look at visible sequelae and the physical attributes as the main causes of stigma has been rendered obsolete [2].

The Explanatory Model Interview Catalog (EMIC) is an adaptable semi-structured interview developed by Weiss and colleagues in response to the challenge of integrating clinical, epidemiological, and social-science frameworks. It was created from open-ended pilot interviews with psychiatric and medical patients in India [14]. Based on Kleinman’s explanatory models, the EMIC incorporates the advances in cultural psychiatry to analyse the relationships between illness-related beliefs and practices, and clinical outcomes [15].

The EMIC interviews have been used to assess stigma in various conditions [16–18]. Questions considered indicators of stigma have been combined into a scale, the EMIC Stigma Scale (EMIC-SS), which is variable in the number of items. According to the condition evaluated and the context of the application, the EMIC-SS can have between 12 [19, 20] up to 17 items [21]. This adaptable scale has been validated in several languages to study stigmas produced by various neglected diseases and conditions, such as Buruli ulcer [22], onchocerchal skin disease [17, 23], leishmaniasis [19], leprosy [21], tuberculosis [2], depression [20], schizophrenia [24], HIV/AIDS [25] and physical disabilities [21].

The EMIC-SS evaluates both the experienced (ES) and the perceived stigma (PS) in one-dimensional [16, 26] as well as two-dimensional models [27]. The ES is the direct stigma suffered by an individual, such as discrimination, rejection, abuse, job loss, or divorce. It occurs when a member of society behaves negatively towards the person affected by a condition. PS refers to the perception, expectation, or fear of experiencing stigma and the awareness of negative attitudes or practices in society [28, 29].

Stigma is the main element that leads to the distress associated with medical conditions and illness behavior [15], so it needs to be systematically assessed and quantified to enable consistent public health policies and to measure interventions against discrimination and prejudice. Generic instruments such as the EMIC-SS are important to measure the extent and severity of stigma, and its change over time [30]. Well validated instruments are essential to compare data from different countries and settings [31]. Several studies have shown satisfactory values of the EMIC-SS construct validity, internal consistency, and test-retest reliability. Rensen *et al* [21] studying 806 people affected by leprosy in India, found an internal consistency of 0.88, a test–retest reproducibility of 0.70, no floor and ceiling effects, and strong positive correlations between the 17-Likert items EMIC-SS and the Internalised Stigma of Mental Illness (0.70) and the Participation Scales (0.68). Vlassoff et al [17] studied 468 patients with onchocercal skin disease in Cameroon, Ghana, Nigeria, and Uganda, found a Cronbach's alpha of 0.80 in a 13-item EMIC-SS. The 15-item EMIC-SS was validated in 260 adults with non-leprosy physical disabilities in Hong Kong [32], demonstrating good internal consistency (0.897), a moderate correlation (r  =  0.48, p  =  0.001) with the Participation Scale, and good reliability (the person and item reliabilities were 0.74 and 0.90, respectively).

At present, Brazilian researchers do not have a validated tool to quantify leprosy-related stigma. Considering that Brazil is one of the countries with the highest number of leprosy cases, with a high prevalence of disabilities [33], and that the current Global Leprosy Strategy 2016–2020: Accelerating towards a leprosy-free world [34] calls all national leprosy programmes to stop discrimination, it is imperative to quantify stigma due to leprosy.

The EMIC-SS recently underwent a rigorous process of cross-cultural adaptation for the Brazilian population [35], but its psychometric qualities are still unknown. We expect to confirm the internal consistency of its one- and/or two-dimensional structures that has been shown in previous studies [27] using confirmatory factor analysis (CFA) and Chronbach alpha. We also planned to evaluate the construct validity and the reproducibility of the Brazilian Portuguese version of the EMIC-SS.

The risk factors described to promote stigma are similar across cultures, regardless of the prevalence, severity, or type of stigma. Among them, visible deformities and disabilities lead to high levels of both felt stigma and self-stigmatization in persons affected by leprosy [21, 29]. Stigma has also been found associated with misconceptions or poor knowledge about the illness, low educational level, and monthly family income [25, 26]. A high level of stigma has been reported in the unemployed or individuals unable to secure employment and, thus that generate no income [26]. Stereotypes and biases are also common regarding obese individuals [36]. In general, age, educational level, income, physical activity, and BMI have shown a correlation in previous studies of stigma related to various conditions [20, 29, 36].

The consequences of stigma are variable in the person affected by leprosy, ranging from psychosocial dysfunction to isolation, depression [37], and low self-esteem [21]. The prevalence of psychiatric disorders among people affected by leprosy is higher than among the general population. In particular, depression is the most prevalent and may lead to decreased social participation and social exclusion [37]. Social participation restrictions are recognized as the result of the stigmatizing attitudes suffered by the persons affected by leprosy [38]. Previous researches have shown a reciprocal relationship between participation and stigma, where a high percentage of patients report experiencing restriction due to stigma [21, 25]. The opposite is observed in individuals who practice physical activity, that are expected to be more resilient to the negative attitudes suffered [39].

Therefore, we formulated the hypothesis that the EMIC-SS score would have significant moderate to high correlations with theoretically related constructs and measures, namely participation restriction, depression, and low self-esteem to assess the construct validation. Besides, the EMIC-SS scores would have significant low correlations with factors that have been associated with stigma, including the social, demographic, and cultural context, psychosocial, and various characteristics of leprosy [21, 26, 29, 36], such as income, educational level, BMI, and frequency of physical activity, were selected for the discriminant validation of the EMIC-SS and its factors.

## Materials and methods

### Participants

Participants were recruited among the patients treated or under surveillance at the Souza Araújo Outpatient Clinic (ASA), Fiocruz, Rio de Janeiro, a leprosy reference center certified by the Joint Commission International. Persons affected by leprosy aged 18 years or older were included during or after multidrug therapy (MDT). People affected by other stigmatizing diseases (e.g. HIV/AIDS); those with previous MDT (were on leprosy relapse or retreatment) or had been treated for less than 2 months, and people with cognitive impairment were excluded.

The sample size to perform the CFA was calculated according to established criteria that recommend at least 10 participants for each item of the scale for an adequate analysis of the model fit and validation [31, 40, 41]. Thus, considering that the scale is composed of 15 items, a minimum of 150 participants needed to be included. The same sample used in the CFA analysis was used to assess the construct validity. The sample size for the reproducibility analysis depended on the participant’s compliance with the appointment.

### Instruments

#### Inventory Explanatory Model Stigma Scale (EMIC-SS)

The cross-culturally adapted EMIC-SS for persons affected by leprosy in Brazil [35] has 15 questions with four Likert-type options: no (0), not sure (1), possibly yes (2), and yes (3). The answer "yes" indicates a strong signal of stigma therefore, it has assigned the highest value (3 points), except for item 2 that has a reversed score. Thus, the higher the total score obtained by the sum of responses, the more severe the stigma perceived by the person affected by leprosy.

We divided the EMIC-SS into the two factors suggested by Weiss [27] by theoretically separating the items to structure each factor according to the definitions of perceived and experienced stigmas.

#### Participation Scale (P Scale)

The scale has good evidence of validity and reliability [15, 26, 38]. It is composed of 18 items that evaluate social participation and the impact of stigma [38]. The individual ranks his/her participation comparing with "peers", ie. people similar to him/herself in all senses but the disease. The response options are scored as not relevant (0), no problem (0), small problem (1), medium problem (2), large problem (4). High P-Scale scores indicate restrictions on social participation.

#### Rosenberg Self-esteem Scale (RSS)

It evaluates a set of self-esteem and self-acceptance emotions. It consists of 10 statements measuring both positive and negative feelings about the self and a Likert-type scale that ranges from strongly agree (1), to strongly disagree (4). The scale is one-dimensional with the highest scores (maximum of 40) indicating a good self-esteem level. The minimum score possible is 10. The scale was adapted and validated in Brazil [42] and had its factorial structure revised by Hutz and Zanon [43].

#### Beck Depression Inventory (BDI)

Composed of 21 statements and a Likert-type scale that ranges from 0 to 3, the BDI is used to detect depressive symptoms. The total score results from adding each response. A high score is suggestive of depression. The instrument was adapted and validated in Brazil and presented good signs of validity and reliability [44].

#### Socio-demographic questionnaire

Developed specifically for this study, it included age, gender, ethnicity, marital status, education level, occupation, monthly family income, physical activity, and general knowledge about the disease, among other items. Clinical data were obtained from the patient’s file.

### Procedure

Four experienced and trained female researchers interviewed the participants and collected data between September 2016 and April 2018. The persons affected by leprosy, previously selected from the ASA database based on the inclusion criteria, were invited to participate while waiting for his/her consultation at the clinic. After free and informed consent each participant was interviewed in a private room for an average of 40–60 minutes when the socio-demographic questionnaire and the scales were applied. The BDI and RSS were offered to be self-applied, except when the participant was illiterate.

To evaluate the test-retest reliability, the same or different interviewer applied the EMIC-SS at the follow-up visit for patients that were under treatment, or 15–30 days after the first interview to participants that came for their annual visit. Non-compliant participants were reinvited up to 2 times.

### Data analysis

The EMIC-SS dimensionality was evaluated by CFA using the LISREL 7 program [45]. The CFA is a theory-driven technique that allows confirming or refuting theoretical and hypothesized factor structures by comparing the statistical fit of the hypothesized model with the fit of theoretically alternative models [46]. If the hypothesized factor structure fits the data, then the factor structure replicates. If it does not fit the data, modification indices are used to inform the localization of the factor pattern constraints that caused the misfit [47, 48]. Standard indices values were used to evaluate the model fit: Goodness-of-fit (GFI) > 0.90, Adjusted Goodness-of-Fit (AGFI) > 0.95, Non-normal Fit (NNFI) > 0.95, Comparative Fit (CFI) ≥ 0.95, Standardized Chi-square (χ^2^/df) < 3.0, and Root Mean Square Error of Approximation (RMSEA) <0.08. Items were dropped if they had factor loadings (λ) ≤ 0.3 [49]. Missing data were deleted listwise. One and two-dimensional models were tested in all subsequent analyses.

The SPSS 19.0 software was used for descriptive and inferential analysis, considering a 95% confidence level. Internal consistency was also assessed with the use of Cronbach's alpha and was considered acceptable with alpha values greater than or equal to 0.7. The presence of floor and ceiling effects was identified when 15% of the participants or more had the lowest (0) and the highest possible scores for the EMIC-SS and its factors [31].

Construct validity was evaluated by testing convergent and known-groups validity. It was considered sufficient if at least 75% of the results were in correspondence with the hypotheses [31]. Convergent validity was assessed using the Spearman correlation. Considering the non-parametric characteristics of the EMIC-SS and the two factors scores, Kruskal-Wallis and Mann-Whitney tests, for non-binomial and binomial variables, respectively, were used for the known-groups validity and exploratory analyses. Post-hoc analysis was used to define the pairs presenting differences of medians.

Regarding convergent validity, we hypothesized that the EMIC-SS score would be related to restriction on social participation, depressive symptoms, and low self-esteem. Thus, we expected a moderate positive correlation (a Spearman correlation coefficient between 0.4 and 0.8) between EMIC-SS, BDI, and P-Scale total scores, and that a moderate but negative correlation would exist between EMIC-SS and RSS. Moderate to high correlations were also hypothesized for the PS and ES factors of the EMIC-SS. Besides, we hypothesized that the EMIC-SS scores would show a significant low correlation with some related socio-demographic and clinical characteristics consistent with discriminant validity testing: the EMIC-SS would have a positive correlation with age, educational level, and BMI (calculated from self-reported height and weight), while it would have a negative correlation with monthly family income, and frequency of physical activity.

Considering known-groups validity, we expected the EMIC-SS to allow differentiating groups related to socio-demographic aspects, knowledge about leprosy, and clinical variables. For these analyses, we categorized schooling and income into binomial variables. Because of the low correlation between the EMIC-SS and PS factor with age, we collapsed it into 20-year groups to assess the group with the highest EMIC-SS scores. Also, BMI was categorized to evaluate the association of obesity in leprosy patients as a stigma enhancer.

According to previous studies on stigma, we hypothesized that females, the elderly, the participants self-declared as black, the unemployed, those who changed occupation when diagnosed with leprosy, that had low schooling level or low income, and those who practiced little or no physical activity would have higher scores than the other categories of each variable. On the other hand, participants who communicated to others about their disease or had adequate knowledge about leprosy would have EMIC-SS scores in the low range. Regarding the clinical characteristics, we expected participants with a disability or visible deformities, complications, or that had BMI within the obesity range, would have higher scores than other groups in the variable.

Finally, regarding reproducibility, we assessed the test-retest reliability using the type C intra-class correlation (ICC) for single measures and the two-way mixed-effects model where people effects are random and measures effects are fixed. Reliability was considered acceptable with an ICC agreement of at least 0.70 within the 95% confidence interval [50].

### Ethical issues

The Oswaldo Cruz Foundation/Fiocruz Institutional Review Board approved the study (Study Registry—CAAE 50625615.9.0000.5248) and Mitchell G. Weiss, the first author of the original scale [3], granted us authorization for the cultural adaptation and validation of the EMIC-SS in Brazil. It is important to note that, following the Brazilian regulation for human research, the participants voluntarily accepted to participate and received no payment for their participation.

## Results

### General characteristics of the sample

**Fig 1:**
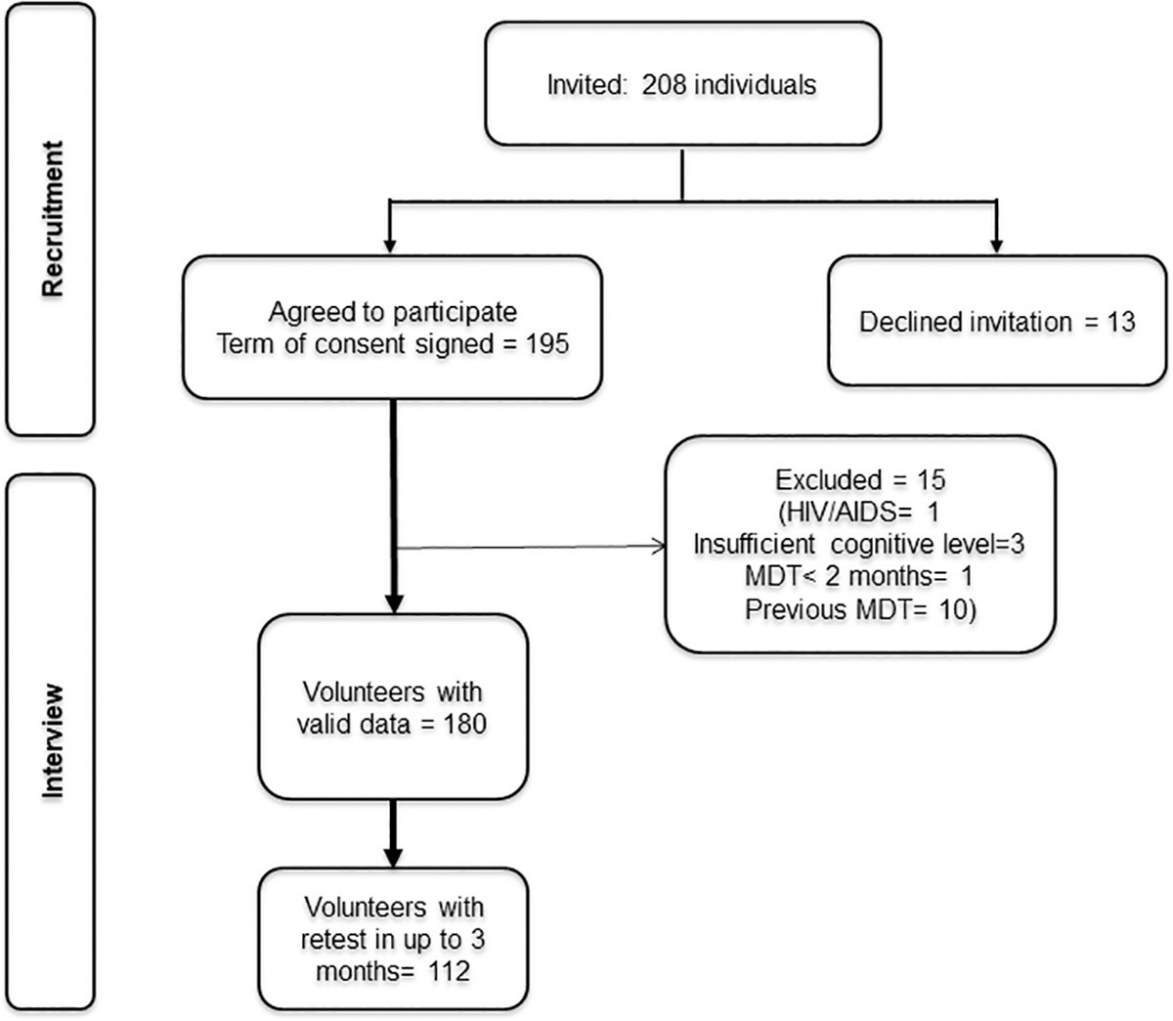
Recruitment and inclusion process flowgram. MDT = multidrug therapy.

A total of 208 individuals were invited to participate, 13 declined the invitation mainly due to lack of time, and 15 people were excluded (Fig 1).

The final sample consisted of 180 individuals, between 18 and 89 years of age, mean age of 49.3 years (SD = 15.14). The participants lived in Rio de Janeiro and surrounding cities but came from States of the five regions of Brazil (Table 1). A high proportion of cases (55.7%) had less than 10 years of schooling (median = 9, min = 0, max = 16). Mean Body Mass Index (BMI) was 26.89 ± 4.82 Kg/m^2^, and 21% of the participants were classified as obese (BMI ≥ 30).

**Table 1:** Socio-demographic and clinical characteristics of the 180 persons affected by leprosy.

Socio-demographic characteristics			Number of cases	Percentage
Sex	Male		123	68.3%
Self-declared skin color	white		47	26.1%
	black		35	19.4%
	brown		80	44.4%
	yellow		5	2.8%
	other		13	7.2%
Place of birth (Brazilian region)	Southeast		136	75.5%
	Northeast		38	21%
	South		3	2%
	Central West		2	1%
	North		1	0.5%
Employment status	Employed		85	47.2%
	Unemployed		19	10.6%
	sickness aid/retired		59	32.8%
	Other (student, housewife)		17	10.4%
Marital status	With partner		117	65%
	Without partner		63	35%
Number of people in household	1 person		17	9.4%
	2–4 people		137	76.1%
	5–7 people		25	13.9%
	8–10 people		1	0.6%
Monthly family income	< 1salary		28	15.6%
	1–2 salaries		102	56.7%
	3–5 salaries		36	20.0%
	>5 salaries		14	7.8%
Physical activity	No activity		134	74.8%
	once/week		3	1.7%
	2–3 times/week		21	11.8%
	4–6 times/week		15	8.4%
	Daily		6	3.3%
**Clinical characteristics**				
Leprosy clinical form	Indeterminate		5	2.7%
	Tuberculoid		2	1.1%
	Borderline tuberculoid		24	13.3%
	Bordeline borderline		19	10.5%
	Borderline lepromatous		41	22.7%
	Lepromatous		80	44.4%
	Pure neural		5	2.7%
	Not informed		4	-
Treatment status	During MDT		62	34.4%
	Follow-up after RMDT with complications and/or sequelae		70	38.9%
	Follow-up after RMDT without complications/sequelae		48	26.7%
Grade of disability at diagnosis	0 (No disability)		92	51.1%
	1 (anaesthesia/paresia)		59	32.8%
	2 (deformity/ulcer)		29	16.1%
Grade of disability during interview	0 (No disability)		94	52.2%
	1 (anaesthesia/paresia)		61	33.9%
	2 (deformity/ulcer)		25	13.9%
Complications^a^	leprosy reaction		42	74%
(n = 58, 32.2%)	neuropathic pain		12	21%
	neuropathy		6	11%
	Not informed		1	-
Co-morbidities^a^			81	45.5%

NA = not applied; MDT = multidrug therapy; RMDT = released from MDT.

^a^Participants could have more than one complication/co-morbidity

### Internal consistency

#### Confirmatory factor analysis

Due to the absence of multivariate normality, the factor model was estimated by the unweighted least squares method. Based on previous studies [16–18], we performed the CFA on the 15 items in two hypothesized factorial models: (1) one-dimensional model and (2) two-dimensional model composed of the factor that evaluates PS (items 1, 2, 3, 4, 5 and 14) and the factor that studies ES (items 6, 7, 8, 9, 10, 11, 12, 13 and 15).

The one-dimensional model did not provide an adequate fit to the data (χ^2^/df = 2.18, RMSEA = 0.082, GFI = 0.94, AGFI = 0.92, NNFI = 0.94, CFI = 0.95). Only the CFI was at cutoff for acceptable fit. Items 1 (λ = 0.291), 2 (λ = -0.075) and 12 (λ = 0.244) had low factor loading and were dropped. The new model retained 12 items and provided a good fit to the data (χ^2^/df = 2.02, RMSEA = 0.076, GFI = 0.96, AGFI = 0.95, NNFI = 0.99, CFI = 0.99). All of the items included had their factorial loads ranging from λ = 0.368 to 0.651 (Table 2). Thus, we confirmed the factor structure of the 12-item EMIC-SS one-dimensional model.

**Table 2:** Internal consistency of the Explanatory Model Interview Catalog-Stigma Scale (EMIC-SS) tested in 180 individuals affected by leprosy.

Factor		Question. English original [Portuguese transculturally adapted]	α	TM (λ)	UM (λ)
**Perceived Stigma**	3	Do you think less of yourself because of this problem? Has it reduced your pride or self-respect? [*Você se considera inferior por causa desta doença*, *ou seja*, *ela diminui seu orgulho ou respeito próprio*?]		0.681	0.646
	4	Have you ever been made to feel ashamed or embarrassed because of this problem? [*Já houve alguma situação que fez você se sentir envergonhado(a) ou constrangido(a) por causa desta doença*?]		0.479	0.455
	5	Do your neighbors, colleagues or others in your community have less respect for you because of this problem? [*Seus vizinhos*, *colegas ou outras pessoas de seu meio social demonstram menos respeito por você por causa desta doença*?]	0.63	0.615	0.593
	14	Have you decided on your own to stay away from work or social group? [*Você decidiu por conta própria ficar afastado(a) de seu trabalho ou de grupos sociais por causa da doença*?]		0.404	0.383
**Experienced Stigma**	6	Do you think that contact with you might have any bad effects on others around you even after you have been treated? [*Você acha que o contato das pessoas com você poderia ter algum efeito prejudicial para elas*, *mesmo após seu tratamento*?]		0.382	0.375
	7	Do you feel others have avoided you because of this problem? [*Você sente que as pessoas têm te evitado por causa desta doença*?*]*		0.659	0.651
	8	Would some people refuse to visit your home because of this condition even after you have been treated? [*Alguém se recusaria a ir a sua casa por causa de sua doença*, *mesmo após seu tratamento*?]		0.536	0.527
	9	If they knew about it would your neighbors, colleagues or others in your community think less of your family because of your problem? [*Se seus vizinhos*, *colegas ou outras pessoas de seu meio social soubessem sobre sua doença*, *eles poderiam desvalorizar sua família por isso*?]	0.71	0.374	0.368
	10	Do you feel that your problem might cause social problems for your children in the community? [*Você sente que sua doença poderia trazer problemas para a vida social de seus filhos ou familiares*?]		0.419	0.412
	11	11a. Do you feel that this disease has caused, or will cause, problems for you to get married? (Unmarried only) [*Você sente que esta doença causou ou causará dificuldades para você ter um relacionamento amoroso*? *(Apenas para pessoas sem parceiro(a))*]		0.398	0.393
		11b. Do you feel that this disease has caused problems in your marriage? (Married only) [*Você sente que esta doença causa problemas em seu relacionamento amoroso*? *(Apenas para pessoas com parceiro(a))*]		0.421	0.415
	13	Have you been asked to stay away from work or social groups? [*Já pediram a você para ficar afastado(a) de seu trabalho ou de grupos sociais por ter hanseníase*?]			
	15	Because of leprosy, do people think you also have other health problems? [*A****s*** *pessoas acham que por você ter hanseníase também tem outros problemas de saúde*?]		0.591	0.580
**Dropped items**	1	If possible, would you prefer to keep people from knowing about your leprosy? [*Se possível*, *você preferiria evitar que as pessoas soubessem que você tem ou teve hanseníase*?]	NA	0.299	0.291
	2^a^	Have you discussed this problem with the person you consider closest to you, the one whom you usually feel you can talk to most easily? [*Você já conversou sobre sua hanseníase com a pessoa que você considera mais próxima de você*, *ou seja*, *aquela com quem você se sente mais à vontade para falar*?*]*		-0.082	-0.075
	12	Do you feel that this disease makes it difficult for someone else in your family to marry? [*Você sente que esta doença dificulta que alguém de sua família tenha um relacionamento amoroso com outra pessoa*?*]*		0.251	0.244

α = Cronbach alpha coefficient, λ = Factor load, SD = standard deviation, TM = two-dimensional model, UM = unidimensional model, NA = not applied

The 15 items correspond to the published English [51] and Portuguese [35] versions of the scale. All questions had minimum values = 0 (no) and maximum values = 3 (yes).

^a^Question 2 has a reversed code.

The two-dimensional model also did not provide an adequate fit to the data in the analysis of the whole scale (χ^2^/df = 2.18, RMSEA = 0.081, GFI = 0.94, AGFI = 0.92, NNFI = 0.94, CFI = 0.95). Like the one-dimensional scale, the RMSEA values were above cutoff and GFI, AGFI and NNFI were below cut-off for acceptance. Items 1, 2 and 12 had low factor loading (λ = 0.299, -0.082 and 0.251, respectively), and thus, they were dropped. The new two-dimensional model provided a good fit to the data (χ2/df = 2.06, RMSEA = 0.077, GFI = 0.97, AGFI = 0.95, NNFI = 0.99, CFI = 0.99) and its factor structure was also co-signed. All of the 12 items (PS factor = 4 items; ES factor = 8 items) had their factorial loads ranging from λ = 0.374 to λ = 0.681 (Table 2). After the exclusion of the three problematic items, the one and two-dimensional models had their factorial structures confirmed.

**Fig 2:**
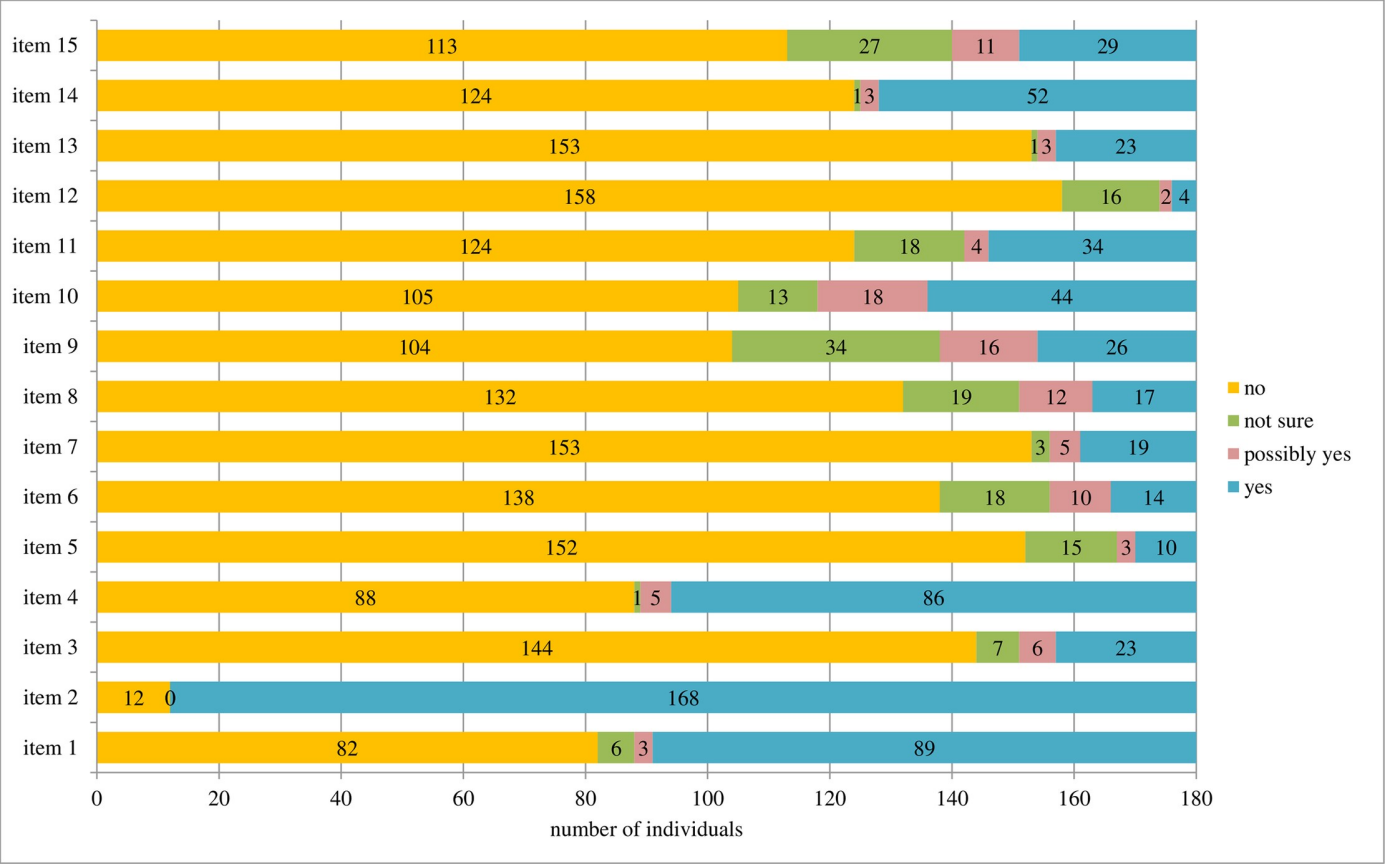
Answer profile to each item of the Explanatory Model Interview Catalog -Stigma Scale by 180 persons affected by leprosy. 0 = no, 1 = not sure, 2 = possibly yes and 3 = yes. NB. Question 2 has a reversed score.

Regarding these problematic items, item 2, had only yes (0) or no (3) answers by the 180 participants (Fig 2). Items 2 and 12 had the highest proportion of 0 responses, 93.3% and 85%, respectively, although most of the items had a no (0) answer, except for items 1 and 4.

### Cronbach alpha

The one and two-dimensional 12-item models showed variable internal consistency indices (Table 2). The EMIC-SS total score and the ES factor had good internal consistency, Chronbachs’s alphas of 0.78 and 0.71, respectively, the PS factor, on the other hand, had a moderate internal consistency (α = 0.63).

### Floor and ceiling effect

No floor or ceiling effects were identified for the whole scale: One (0.6%) of the participants had the highest possible score of 36 in the EMIC-SS while 10.6% of the participants scored 0. No ceiling effect was observed for either of the factors, 2.2% of the participants scored 12, the maximum score in the PS, while 1.1% of the participants scored 24, the maximum score in the ES. However, in both the factors a floor effect was identified, since 38% and 20.1% of the participants had the lowest possible score of 0 in the PS and the ES, respectively.

The next steps in the data analysis were to evaluate the reliability and the convergent and known-groups validities. All the analyses included both the total score of the scale (one-dimensional model) and the scores by each one of the factors (two-dimensional model).

### Construct validity

Two measures of construct validity were evaluated: convergent and known-groups validity. Three scales that evaluate constructs related to stigma manifestations, such as social participation, depression, and self-esteem were used for convergent validity (Table 3). RSS and BDI were self-applied in a few cases, 17 (9.7%) and 24 (13.9%) participants, respectively.

**Table 3:** Descriptive analysis of the scales used for convergent validation of the 12-item Explanatory Model Interview Catalog -Stigma Scale (EMIC-SS): Beck depression inventory, Social Participation Scale, and Rosenberg Self-esteem Scale.

		EMIC-SS	PS factor	ES factor	BDI	P Scale	RSS
		n = 180	n = 180	n = 180	n = 178^a^	n = 173^a^	n = 169^a^
Minimum		0.00	0.00	0.00	0.00	0.00	11.00
Maximum		36.00	12.00	24.00	45.00	60.00	40.00
	25	3.00	0.00	1.00	5.00	3.00	32.00
Percentiles	50 (Median)	6.00	3.00	4.00	10.00	7.00	35.00
	75	12.00	6.00	7.00	15.00	12.00	38.00
	95	23.00	9.00	15.00	31.00	30.00	40.00

Explanatory Model Interview Catalog-Stigma Scale (EMIC-SS) PS = perceived stigma, ES = experienced stigma, Beck depression inventory (BDI), Social Participation Scale (P Scale) and Rosenberg Self-esteem Scale (RSS), n = sample size.

^a^n < 180 due to missing values that were excluded listwise for analysis

### Convergent and discriminant validities

The correlations observed between the scales compared confirmed the *a priori* hypothesis (Tables 4 and 5). Moderate positive correlations were observed between the total scores of the 12-item EMIC-SS when compared with the BDI and P Scale, and a moderate negative correlation was found with the RSS scores (Table 4). The PS and ES factors had moderate positive correlations with BDI scores, but low correlations with P Scale and RSS scores. The total score and the PS factor showed a low and negative correlation with age (rs = -0.224 and -0.240, respectively). ES factor also showed a very low and negative correlation with monthly family income (rs = - 0.165). The other variables evaluated, educational level, BMI, and the frequency of physical activity showed no correlation with the EMIC-SS or its factors (Table 5).

**Table 4:** Construct validity of the 12-item Explanatory Model Interview Catalog -Stigma Scale (EMIC-SS) tested in 180 participants affected by leprosy (Spearman’s correlation coefficients): Spearman’s correlation coefficients of EMIC-SS and its two factors in comparison with the 3 scales.

	PS factor	ES factor	BDI	P Scale	RSS^a^
EMIC-SS	0.795**	0.873**	0.513**	0.448**	-0.413**
PS factor	-	0.439**	0.473**	0.396**	-0.397**
ES factor		-	0.425**	0.372**	-0.396**
BDI			-	0.554**	-0.500**
P Scale				-	-0.461**

EMIC-SS **=** Explanatory Model Interview Catalog -Stigma Scale, PS = perceived stigma, ES = experienced stigma, BDI = Beck depression inventory, P Scale = social Participation Scale, RSS = Rosenberg Self-esteem Scale.

**Correlation is significant at the 0.001 level (1-tailed).

^a^Coefficients are negative due to inverse relation of the scores. High RSS scores indicate high self-steem while high EMIC-SS scores indicate low stigma.

**Table 5:** Construct validity of the 12-item Explanatory Model Interview Catalog -Stigma Scale (EMIC-SS) tested in 180 participants affected by leprosy (Spearman’s correlation coefficients): Spearman’s correlation coefficients of EMIC-SS and its two factors in comparison to socio-demographic characteristics.

	Age	EL	MFI	BMI^a^	PA^b^
EMIC-SS	-0.224*	-0.043	-0.142	0.053	0.001
PS factor	-0.240**	0.036	-0.047	-0.062	-0.114
ES factor	-0.132	-0.106	-0.165*	0.131	0.089
Age	-	-0.253**	0.012	0.140	0.235
EL		-	0.392**	-0.068	-0.122
MFI			-	-0.049	0.027
BMI				-	0.076

EMIC-SS **=** Explanatory Model Interview Catalog-Stigma Scale, PS = perceived stigma, ES = experienced stigma, EL = education level (years of schooling), MFI = Monthly family income, BMI = body mass index, PA = frequency of physical activity.

*Correlation is significant at the 0.05 level (2-tailed)

**Correlation is significant at the 0.001 level (2-tailed).

a. Data for 139 participants

b. Data for 45 participants that referred practicing physical activity.

Given that all of the correlations of the EMIC-SS with the instruments were in correspondence with the hypotheses we confirmed its convergence validity.

### Known-groups validity

For known-groups validity, we verified if the 12-item EMIC-SS and its two factors could differentiate groups concerning socio-demographic aspects, knowledge about leprosy, and clinical variables (Tables 6–8). The EMIC-SS and the ES factor allowed discriminating groups related to age, change of occupation after leprosy diagnosis, monthly family income, and communicating about leprosy diagnosis to others. The PS factor only discriminated according to age categories (Table 6).

**Table 6:** Known-groups validity of the 12-item EMIC-SS based on socio-demographic characteristics.

Characterístics	N	EMIC-SS score		PS fator score		ES fator score	
		**Mean rank**	**(p-value)**	**Mean rank**	**(p-value)**	**Mean rank**	**(p-value)**
**Gender**	**180**		(0.114)		(0.129)		(0.182)
Female	57	99.48		98.88		98.04	
Male	123	86.34		86.62		87.00	
**Self-declared skin color**	**180**		(0.083)		(0.380)		(0.143)
White	47	78.56		81.29		79.77	
Black	35	102.03		100.84		98.24	
Brown	80	88.26		91.01		89.03	
Other	18	109.19		92.19		110.00	
**Age**	**180**		(0.002)*		(0.005)*		(0.006)*
18–39 years old	57	98.67	(0.003)*	103.07	(0.002)*	92.04	(0.046)*
40–59 years old	71	99.65	(0.001)**	93.70	(0.017)*	102.55	(0.001)**
≥60 years old	52	69.05^a^		72.35^a^		72.36^a^	
**Birthplace**	**180**		(0.110)		(0.240)		(0.181)
Southeast/South	139	87.14		88.10		87.75	
Northeast/North/Central West	41	101.90		98.62		100.00	
**Schooling**	**176**		(0.688)		(0.915)		(0.337)
Elementary (<10 years of schooling)	98	89.87		88.15		91.77	
Secondary and above	78	86.78		88.94		84.39	
**Employment Status**	**179**		(0.137)		(0.159)		(0.425)
Employed	85	89.53		90.67		88.11	
Sickness aid/retired	59	87.92		85.93		91.52	
Unemployed	19	114.95		113.42		107.05	
Other (student, housewife)	17	77.00		79.88		80.44	
**Income**	**180**		(0.048)*		(0.412)		(0.025)*
Low (≤ 2 salaries)	28	95.25		92.41		95.88	
Medium-high (3 or more salaries)	50	78.16		85.54		76.52	
**Change of occupation after diagnosis**	**179**		(0.039)*		(0.359)		(0.021)*
No	171	88.27		89.26		88.08	
Yes	8	126.94		105.88		131.13	
**Told others about the disease**	**178**		(0.032)*		(0.250)		(0.013)*
Only family	49	96.77	(0.032)*	89.40		98.79	(0.012)*
Only family, friends, and/or people at work	70	96.49	(0.018)*	96.24		96.48	(0.010)*
Everyone	59	75.18^b^		81.58		73.51^b^	
**Knows someone with leprosy**	**179**		(0.589)		(0.610)		(0.699)
No	93	87.99		88.16		88.57	
Yes	86	92.17		91.99		91.55	
**Practices physical activity**	**180**		(0.827)		(0.592)		(0.849)
No	134	91.00		91.68		90.93	
Yes	46	89.05		87.07		89.25	

n = sample size, EMIC-SS = Explanatory Model Interview Catalog-Stigma Scale, PS = perceived stigma, ES = experienced stigma

*Significant at the 0.05 level (2-tailed)

**Significant at the 0.001 level (2-tailed)

**NB.** Non-parametric tests were used for 2- or k-independent samples (the Mann-Whitney U and Kruskal-Wallis tests, respectively), according to the number of categories in each group. **Post-hoc analysis** (Mann-Whitney U-test): a = significant difference between ≥ 60 years old and other age groups; b = significant difference between participants that referred telling everyone about their disease and those that told only to family members or to family members and friends or people at work.

**Table 7:** Known-groups validity of the 12-item EMIC-SS based on knowledge about leprosy.

Characteristics	n	EMIC-SS score		PS fator score		ES fator score	
		**Mean rank**	**(p-value)**	**Mean rank**	**(p-value)**	**Mean rank**	**(p-value)**
**Searches for information about the disease**	**180**		(0.233)		**(0.023)**		(0.990)
No	66	84.43		79.30		90.56	
Yes	114	94.01		96.98		90.46	
**Refers knowing the cause of leprosy**	**180**		(0.546)		(0.164)		(0.906)
No	104	88.50		86.03		90.89	
Yes	76	93.24		96.61		89.97	
**Knows the correct cause**	**76**		(0.362)		(0.570)		(0.423)
No	9	44.78		43.33		44.00	
Yes	67	37.66		37.99		37.76	
**Refers knowing ways of transmission**	**180**		(0.987)		(0.382)		(0.651)
No	67	90.58		87.24		92.77	
Yes	113	90.45		93.03		89.15	
**Knows the correct way of transmission**	**109**		(0.859)		(0.968)		(0.816)
No	88	55.26		54.94		55.34	
Yes	21	53.90		55.24		53.57	
**Knowledge about leprosy signs and symptoms**	**179**		(0.020)*		(0.120)		(0.028)*
Ignores or knows only one	29	69.52		76.78		70.76	
Knows 2 or more signs and/or symptoms	150	93.96		92.56		93.72	
**Knows prescribed treatment**	**117**		(0.498)		(0.857)		(0.219)
No	32	62.45		58.11		65.25	
Yes	85	57.70		59.34		56.65	

n = sample size, EMIC-SS **=** Explanatory Model Interview Catalog-Stigma Scale, PS = perceived stigma, ES = experienced stigma

**NB.** The non-parametric Mann-Whitney U test was used for 2—independent samples.

**Table 8:** Known-groups validity of the EMIC-SS (12 items) based on Clinical data.

Characteristics	n	EMIC-SS score		PS factor		ES factor	
		**Mean rank**	**(p-value)**	**Mean rank**	**(p-value)**	**Mean rank**	**(p-value)**
**Treatment status**	**180**		(0.848)		(0.794)		(0.666)
During MDT	62	89.61		87.08		91.17	
Follow-up after RMDT with complications/sequelae	70	93.14		92.91		93.67	
Follow-up after RMDT without complications/sequelae	48	87.79		91.41		85.01	
**Had difficulties handling the treatment**	**180**		**(<0.001)**		**(<0.001)**		**(<0.001)**
No	152	82.51		84.00		83.20	
Yes	28	133.88		125.79		130.14	
**Leprosy complications on day of interview**	**179**		(0.117)		(0.365)		(0.072)
No	122	85.87		87.68		85.27	
Yes	57	98.84		94.96		100.13	
**Grade of physical disability on leprosy diagnosis**	**180**		(0.346)		(0.381)		(0.323)
0 (no disability)	92	92.65		93.39		91.11	
1 (anaesthesia/paresia)	59	93.47		91.79		95.64	
2 (with deformities/ulcers)	29	77.66		78.72		78.10	
**Grade of physical disability on day of interview ± 3 months**	**180**		(0.365)		(0.947)		(0.139)
0 (no disability)	94	87.56		90.05		86.90	
1 (anaesthesia/paresia)	61	98.01		92.05		100.61	
2 (with deformities/ulcers)	25	83.24		88.40		79.34	
**Body mass index**	**139**		(0.673)		(0.341)		(0.497)
(<25 Kg/m^2^ (normal/underweight)	51	70.28		75.17		66.11	
25–29 Kg/m^2^ (overweight)	59	67.17		64.50		69.87	
≥30 Kg/m^2^ (obese)	29	75.26		72.10		77.10	

n = sample size, EMIC-SS **=** Explanatory Model Interview Catalog-Stigma Scale, PS = perceived stigma, ES = experienced stigma

The median scores of the 12-item EMIC-SS and the factors were significantly different between the three age groups: χ^2^ = 12.558 (p value = 0.002) for the EMIC-SS, χ^2^ = 10.603 (p value = 0.005) for the PS factor, and χ^2^ = 10.290 (p value = 0.006) for the ES factor. Participants 60 years old or more had a significantly lower scores (medians of 3.5, 3.0 and 0 for the EMIC-SS, ES and PS, respectively) than participants between 18–40 (medians of 7.0, 4.0, and 3.0) and 41–59 years old (medians of 7.5, 5.0 and 3.0).

As expected, participants that changed occupation when diagnosed with leprosy had a significantly higher score (median EMIC-SS score = 13) than those who remained at their jobs (median EMIC-SS score = 6). Likewise, participants with a low income had higher scores (median EMIC-SS score = 6.5) when compared to those with higher family income (median EMIC-SS score = 5.5).

All of the participants, but two, mentioned having told about their diagnosis to someone. Nevertheless, 12 participants denied discussing the problem with the person closest to them at the EMIC-SS interview (answered “no” to item 2, Fig 2). Those who told everyone about their disease (median EMIC-SS score = 5) also had significantly lower scores than those who only told to family members (median EMIC-SS score = 7), or to family and friends or people at work (median EMIC-SS score = 6.5).

Out of the 76 participants that referred to knowing what caused leprosy, 88.2% told the exact cause: bacteria, bacilli, or microorganism. Asked about how they get the disease, 62.8% said they knew, but in fact, only 4.9% knew the correct form of the transmission. For 68% of the cases, the transmission was via the air or wind, which we considered a wrong answer. The scales did not allow discriminating groups about this knowledge. Nevertheless, EMIC-SS and the ES factor discriminated between knowing or not about signs and symptoms (Table 7). Among the symptoms more frequently cited by the participants were skin patches (37.8%) and numbness (32.9%). Interestingly, six participants referred to fear, sadness, shame, isolation, or depression. Participants who indicated several symptoms of the disease had higher scores (median = 6 and 4, for EMIC-SS and ES factor, respectively) than participants who were unable to cite any symptoms or only one (median = 3 and 2, for EMIC-SS and EF factor, respectively). In addition, the PS factor distinguished between participants that searched for information about the disease and those who did not. Participants referred to looking for information only at the media (40.4%), only at the health center (28%) and only from acquaintances (4.3%), or in all of them (27.2%). Surprisingly, those who referred to not searching for information had significantly lower scores (median = 1) than the participants who referred to looking for information (median = 3).

Regarding the clinical data, the scale and its factors discriminated only between participants with difficulties in handling his/her treatment (Table 8). People who referred finding the treatment difficult had significantly higher (p value < 0.001) EMIC-SS scores (median = 14), PS factor scores (median = 6) and ES factor scores (median = 9) than those who did not (EMIC-SS median = 6 and PS and ES factors median = 3).

### Reproducibility

To determine the reproducibility of the EMIC-SS, we evaluated the stability measure of the test applied on two different occasions in the same person. A total of 112 (62%) participants returned to the clinic less than three months after the day of inclusion. The retest was applied at a mean of 36.5 ± 18.2 days after the first interview. We expected this period to be long enough to prevent recall, and short enough to ensure that the participants have had no significant clinical change.

Only 21.4% of the participants maintained the same score on the 12-item scale. However, a good reliability was observed indicated by the test-retest ICC of 0.751 (95% Confidence Interval = 0.657–0.822, p < 0.0001) for the 12-item scale. For the ES factor, a just above cut-off ICC value of 0.702 (95% Confidence Interval = 0.595–0.785, p < 0.0001) was observed, and an insufficient ICC for the PS factor, 0.636 (95% Confidence Interval = 0.511–0.735, p < 0.0001).

## Discussion

The Global leprosy strategy 2016–2020: accelerating towards a leprosy-free world [24] calls for action to reduce stigma and discrimination and to promote the inclusion of persons affected by leprosy. This study evaluates the factor structure, validity, and reliability of EMIC-SS applied to persons affected by leprosy in Brazil, making available an important tool for researchers to allow the production of information about the prevalence of social stigma in the country. It can be an aid to obtain data for monitoring stigma in Brazil, which is an essential indicator of the third pillar of the strategy: “Stop discrimination and promote inclusion”.

Our results demonstrated that the scale is a valid and reliable measure of stigma in the sample studied. The factorial structures of the two models fit the observed data after reducing the number of 15 items to 12. It is worth remembering that the elimination of items presenting problems in their structure is one of the basic assumptions in the instrument validation process [40, 41]. This is an accepted procedure in scale validation studies [52]. Other authors have decided to retain or drop items from the EMIC only guided by the Cronbach alpha statistic [16–18]. The selection of items in the scale based on statistical criteria should take into consideration how it affects the measurement properties of the instrument. However, the Stigma Scale obtained from the semistructured EMIC inter-view is not a fixed scale, so its component items can differ in both number and type in the different studies according to the population and condition under evaluation.

It is interesting to note that the three problematic items were the same in both models. Several hypotheses may warrant such findings. In the first place, the content of these three items may not have been sensitive to the stigma assessment in the sample and, therefore, did not present good adjustments in the models tested. Another hypothesis would be related particularly to the inability of items 1 and 2 to discriminate groups. These two items were also considered problematic in a validation study for people with non-leprosy disabilities [32]. Although frequently patients omit that they are affected by leprosy, as reported in Northeast of Brazil [53] where 74.8% of 107 participants had not disclosed their condition to others, our study pointed out that only 2 of the 180 participants concealed this information. This fact may have influenced the dropping of item 1, which seeks to know the potential for the omission of informing others about the disease, and item 2, which evaluates the interviewee's willingness to talk about the disease with close contacts. The third hypothesis is related to the cross-cultural adaptation. Some of the items translated into Portuguese may have used words or phrasal structures that are not sufficiently clear for the reality of the population. This may have been particularly the case of item 12, which raised doubts regarding the term "to have a love relationship with another person" that was used instead of the original “to marry”. Many couples in Brazil live together as partners without a formal marriage, but it was common for participants to understand the question as loving oneself or to ask for an explanation. Specifically regarding item 2, the question structure only allows a yes or no answer. Thus, this item could be separated from the scoring calculation, but still can be maintained as part of the instrument.

The internal consistency of the scale and its factors was moderate. The PS factor had the lowest alpha coefficient, probably due to the small number of items included in this factor. The alpha coefficient is proportional to the number of items in a scale or factor. Scales with many items are more reliable and have high alpha values. Thus, the one-dimensional model of the EMIC-SS with the highest number of items (12 items) tends to be more reliable (with greater internal consistency) than the two-dimensional model in the evaluation of stigma. The alpha coefficient for the one-dimensional model approached values reported in previous studies [16, 21].

We hypothesized that the EMIC-SS could be associated with theoretically related constructs such as depression, restriction of social participation, and low self-esteem. Thus, the BDI, P Scale, and RSS, three well-known validated instruments, were used for convergent validity analysis. As hypothesized, the total scores of the scale and its factors had moderate positive monotonic correlations with depression and restriction in social participation, and negative monotonic correlations with self-esteem, confirming the convergent validity for the one and two-dimensional models.

Other hypothesized constructs such as age, educational level, income, physical activity, and BMI have shown a correlation in previous studies of stigma related to various conditions [20, 29, 38, 53–55]. We observed a weak negative monotonic correlation of the EMIC-SS and the PS factor scores and age. However, no association was established with educational level, income, frequency of physical activity, nor with BMI. The Quetelet Index (kg/m2) was calculated from self-reported height and weight, which may be inaccurate. But in a study about teen obesity, the authors recommended the use of the self-reported parameters to study obesity [56]. However, they reported that weight perception in teens and their parents was not related to BMI. Discrepancies between image perception regarding weight perception and BMI are common. In obese individuals recovering normal body mass, the effects of stigma persist regardless of the body mass [39].

By looking at different factors previously reported to be associated with leprosy-related stigma such as the socio-demographic and clinical aspects, and the participants’ knowledge about leprosy, we showed that the EMIC-SS and its factors were able to discriminate several groups. Age groups presented significant differences in the three scores. The scores of participants from 18–39 years old were significantly higher than those of elderly participants, as shown by Shi-Jie *et al*. [20]. However, other authors have reported the association of stigma with older-age [57] or no significant differences with age [58]. Even though the contradictions found in the literature, the results suggest the validity of the scale and its factors discriminating age groups.

Leprosy commonly affects less favored social classes, reduces the quality of life and work capacity of the affected person. These factors may contribute to perpetuating leprosy-associated stigma [24]. Accordingly, low socioeconomic status has been associated with stigma [29, 58]. Similarly, in our sample, the EMIC-SS and the ES factor discriminated groups regarding the monthly family income.

The EMIC-SS did not discriminate groups related to employment status. Although unemployed participants had higher scores on the scale and its factors, they were not significantly higher than employed participants or other groups. Previous studies report that unemployment is an important risk factor for negative attitudes towards people with leprosy [29, 58]. Besides, a recent study in China [20] showed significant differences in stigma scores between retired people affected by leprosy and full-employed participants.

Consistent with Adhikari *et al*. [59], which found high stigma among participants in Nepal who changed their occupation after diagnosis, our findings also revealed that participants who reported having changed their occupation after receiving the diagnosis of the disease presented higher scores on the scale and the ES factor. It is another indication of the known-groups validity of the scale.

A clear sign of stigma is hiding the diagnosis of leprosy [53]. Only ES factor discriminated group concerning telling others about leprosy. The omission is a cultural pattern of confrontation or even a defence mechanism against negative conceptions of society. The ES factor is especially sensitive to the experiences of discrimination suffered, which would lead to avoiding telling others about the disease.

Traditional beliefs and lack of knowledge may lead to the persistence of stigma. Among the several variables related to knowledge about leprosy, we observed that the EMIC-SS or its factors discriminated groups only regarding the search for information about the disease and being able to name signs and symptoms of leprosy. The participants mostly named their symptoms which are related to their own experience of illness. In this way, their self-perception of the disease could be reflected in the higher ES factor scores observed in this group of participants in comparison to those who were not able to identify signs or symptoms. It may also explain why only ES factor that reflects the direct stigma suffered by the individual, and not the PS factor, was able to discriminate between those who knew from those who did not know the signs and symptoms. Interventions to address stigma in leprosy endemic countries such as Thailand and Nepal have proven effective when including self-care practice, tailored-made leprosy information, and promotion of income-generating activities in people affected by leprosy, the community, and health care workers [60, 61]. Most of the people we interviewed had some sort of income, had schooling, and were encouraged to practice self-care. These characteristics may reduce the capacity of EMIC-SS and its factors to discriminate groups in our sample according to their knowledge of leprosy.

Only the PS factor discriminated participants who searched for information from those who did not. At the outpatient clinic, the patients receive information about the disease, the treatment, and possible complications in a program of health education aimed to increase patients’ knowledge regarding the disease, its complications, and sequelae. Most participants referred to searching for information about the disease, mainly in media and with health professionals. PS reflects the fear of the people affected by leprosy of experiencing stigma, which could drive them to look for information about the disease [4].

Regarding the clinical characteristics evaluated, the EMIC-SS and its factors only discriminated between patients that had difficulty in managing their treatment from those who easily handled their treatment. Similarly, Adhikari *et al*. [59] found that persons affected by leprosy who considered it difficult to handle treatment had higher scores in the EMIC as compared to those who did not consider it difficult. It is widely known that delayed treatment and poor adherence to treatment are common in people who experience, perceive, or internalize health-related stigma [4].

EMIC-SS and its factors did not discriminate groups concerning the grade of physical disability and visible deformities. Studies in India [21] and Nepal [59] have found higher stigma perception in individuals with deformity when compared to those without. These contradictory findings could be related to the diverse cultural context of the three countries (Brazil, India, and Nepal). Cross and Choudhary [61] investigated the effect of the Stigma Elimination Program (STEP) on the social participation of persons affected by leprosy aged between 15–65 years, in Nepal using the P Scale as a proxy scale for measuring stigma. The authors found that only 10.5% of the 76 participants with a visible disability that received the intervention had restricted participation, while among the 81 with disability from the non-STEP villages, 38% reported participation restriction. At the outpatient clinic, the patients are followed closely by a multidisciplinary team, with support from a physiotherapist and social worker, as part of the program of prevention of disabilities. Although not focused on stigma, multidisciplinary support may have contributed to the low scores observed in patients with deformities. Thus, interventions with a focus on stigma reduction, such as continuous health education and support groups, accessibility to physical rehabilitation centers, may be useful in making people with deformities more responsive to stigma.

The ability of the EMIC-SS and its factors to differentiate several hypothesized groups (age, change of occupation, monthly family income, communicating others about the disease, search for information about the disease, knowledge about leprosy signs and symptoms, and perception of difficulty to follow treatment) confirms the scale known-groups validity, both in its one and two-dimensional models. It is important to note that most of the groups were related to socio-demographic variables. When comparing the scores of EMIC-SS and PS and ES factors, it was possible to observe that EMIC-SS and ES factor were able to discriminate the same six groups, while the PS factor, only discriminated three groups, one of which was not discriminated by the whole scale nor the ES factor. Again, as for the reliability, the limited PS factor of discriminating groups may be associated with the few numbers of items included in this factor, which was below the recommended value of 5 for a strong and stable factor [37].

The Brazilian Portuguese version of the EMIC-SS was reliable. The strong ICC found between the test and retest ensures the stability of the instrument. Another validation study in India [21] also reported a strong association between test and retest, corroborating the results of this research.

Regarding the total scores of the EMIC-SS, some aspects need consideration. The values observed in the present study were below previous studies that have used the scale. Rensen *et al* [21], in a sample of 806 Indians affected by leprosy, found a mean EMIC score of 13.8 ± 12 (ranging from 0–54); Rafael *et al*. [16], with a sample of 80 people with epilepsy in Benin, found a mean of 21.9 ± 11.8 (ranging from 0–48). The apparent difference in the total scores is related to the different versions of the EMIC-SS that have been used in various cultural contexts. While in our study the models used to analyze the scores resulted from the present validation process, with 12 items in the one and two-dimensional versions, the studies by Rensen *et al*. [21] and Rafael *et al*. [16] used the EMIC-SS with 17 and 15 items respectively, some of which are a different in content. It is worth remembering that the versions of the scales validated in different countries with different number of items make it difficult to compare the scores obtained in cross-cultural studies. Future studies could continue to explore the factorial structure of EMIC-SS in different cultural contexts to obtain more homogeneous versions of the number and type of items of the scale.

Because of the differences in the total scoring and item selection to compose the EMIC-SS in the various settings, the interpretability of the scale scoring is essential. The transformation of the quantitative value is therefore necessary to convey a qualitative meaning to the scores. Since the score is a non-continuous variable, it would be appropriate to use the 25th and 75th percentiles of the 12-item scale score to define the cut-off values to establish the level of stigma. Further studies using the Brazilian cross-culturally adapted EMIC-SS are needed to set up the levels to identify a meaningful change in individual and group evaluations or to compare relevant sub-group of patients. Classifying stigma perception into low, moderate, or high levels can facilitate comparison between groups and populations and assessing the effect of stigma elimination interventions.

Possible limitations of the study should be mentioned regarding the sample and interview methodology. A heterogeneous and representative sample of the target population is recommended for instrument validation [49]. In a continental multicultural country such as Brazil, it is difficult to achieve this ideal in a single-center study. Nevertheless, we recruited participants from the five regions of the country. Although most of them were born in Rio de Janeiro, the State has a history of being attractive to migrations from other Brazilian States and countries. The metropolitan area of Rio de Janeiro concentrated most of the State population between 1900 and 1970. First, as the country’s capital then because of industrialization, it has always attracted a great diversity of interstate migrations [62], which renders the population heterogeneous. Therefore, we understand that there is little sampling bias in this exploratory study.

It is usually recommended that data collection procedures be homogeneous and free of bias. Although we had a mixture of interviewer-administrated and self-administrated forms, a small proportion of the RSS and BDI was self-administered. Having the interviewer apply the instruments was necessary since we received people with low education levels and functional illiterates. Our adaptation looked to include these types of participants, despite cognitive limitation due to the high level of illiteracy in the country IBGE and among people affected by leprosy [63]. Besides, the researchers who collected the data were experienced in this type of procedure. Another limitation is inherent in any instrument validation process. Measures intended to be indicators of particular constructs may not be retained [64, 65]. Any scale resulting from a validation study is imperfect since it presents several points that need improvement.

## Conclusions

Stigma measurement is essential to understand the burden stigma and to assess the effectiveness of interventions. Quantitative and qualitative instruments are available, but the EMIC-SS serves as a generic tool to study leprosy-related perceived and self-stigma [51]. We studied the factor structure and the psychometric qualities of an instrument capable of evaluating perceived and experienced stigma in both one-dimensional and two-dimensional models. Our findings indicate that the one-dimensional model presents satisfactory evidence of factorial validity, internal consistency, convergent and known-groups validity, no floor or ceiling effects, and test-retest reliability to evaluate leprosy-related stigma in Brazil. We recommend that further exploration of the factorial structure of EMIC-SS in other samples and populations with stigmatizing conditions to strengthen the indications of the validated scale and to determine the optimal factor structure. We encourage researchers to continue to explore the factorial structure, validity, and reliability of EMIC-SS in different samples and contexts. These types of studies are rare in the field of leprosy, and evaluating a construct as complex as stigma with a single instrument is particularly challenging.
